# Supplementary material for: The genome sequence of the Water Veneer, Acentria ephemerella (Denis & Schiffermüller, 1775)
Source: Wellcome Open Res. 2024 Mar 8;9:134. [Version 1] doi: 10.12688/wellcomeopenres.21099.1 (PMC11109561; doi:10.12688/wellcomeopenres.21099.1)
Supplement: Peer review history

# Reviewer report: Reviewer response for version 1

**DOI:** 10.21956/wellcomeopenres.23339.r80953

**Published:** 2024-05-10

**Authors:** Wai Lok So

**Affiliations:** 1 Center for Soybean Research of the State Key Laboratory of Agrobiotechnology, The Chinese University of Hong Kong, Hong Kong, Hong Kong; 2 School of Life Sciences, The Chinese University of Hong Kong, Hong Kong, Hong Kong

Boyes and Mulhair have generated the genome of a crambid moth,  that is potentially a biocontrol agent towards non-native pest plants. The genome sequenced is of high quality with high BUSCO value and 99.9% reads have been anchored on 31 pseudomolecules that can be regarded as a genome of very good quality. The methods are sound and standard, while the validation processes of the sequenced genome is also valid.

 Considering the background, the significance of generating a genome of this specie and its application or research related to biocontrol agent was not completely elaborated in the  background. It is encouraged to mentioned more about how this sequenced genome can be applied to ecological measures/research.

 Besides, a more detailed description should be stated on how this species identity was confirmed. Is it based on molecular or morphological data? Please kindly provide the identification rationale in the method section.

Are sufficient details of methods and materials provided to allow replication by others?

Yes

Is the rationale for creating the dataset(s) clearly described?

Partly

Are the datasets clearly presented in a useable and accessible format?

Yes

Are the protocols appropriate and is the work technically sound?

Yes

Reviewer Expertise:

genomics, molecular biology, soil biodiversity, arthropods

I confirm that I have read this submission and believe that I have an appropriate level of expertise to confirm that it is of an acceptable scientific standard.

# Reviewer report: Reviewer response for version 1

**DOI:** 10.21956/wellcomeopenres.23339.r80956

**Published:** 2024-05-07

**Authors:** Antónia Monteiro

**Affiliations:** 1 National University of Singapore (Ringgold ID: 37580), Singapore, Singapore

This manuscript provides a chromosome-level assembly and annotation of the nuclear and mitochondrial genomes of a single male of *Acentria ephemerella,* a moth species from the family Crambidae. This is a very interesting species that has aquatic larvae as well as females that are polymorphic for the presence of wings. It provides an excellent system for understanding how a sex-limited polymorphism for presence/absence of wings comes about and to study specific larval adaptations to water.

 The manuscript is straightforward. The sequencing was performed primarily with PacBio single-molecule HiFi long reads (at 64-fold coverage), and the contigs were scaffolded with Hi-C data. The N50 is 12.3 Mb. The assembly has a BUSCO completeness of 98.8%. The availability of this genome should facilitate both functional genetics as well as comparative genomic studies in future.

Are sufficient details of methods and materials provided to allow replication by others?

Yes

Is the rationale for creating the dataset(s) clearly described?

Yes

Are the datasets clearly presented in a useable and accessible format?

Yes

Are the protocols appropriate and is the work technically sound?

Yes

Reviewer Expertise:

evo-devo of lepidopterans

I confirm that I have read this submission and believe that I have an appropriate level of expertise to confirm that it is of an acceptable scientific standard.
